# Myostatin increases the expression of matrix metalloproteinase genes to promote preadipocytes differentiation in pigs

**DOI:** 10.1080/21623945.2022.2065715

**Published:** 2022-04-20

**Authors:** Zhe Zhu, Akhtar Ali, Jing Wang, Shijin Qi, Zaidong Hua, Hongyan Ren, Liping Zhang, Hao Gu, Adrian Molenaar, Masroor Ellahi Babar, Yanzhen Bi

**Affiliations:** a Key Laboratory of Animal Embryo Engineering and Molecular Breeding of Hubei Province, Institute of Animal Science and Veterinary Medicine, Hubei Academy of Agricultural Sciences, Wuhan, Hubei, China; b Department of Biotechnology, Virtual University of Pakistan, Lahore, Pakistan; c College of Life Science, South-Central University for Nationalities, Wuhan, China; d Rumen Microbiology and Animal Nutrition and Physiology AgResearch, Grasslands Campus, Fitzherbert Research Centre, Palmerston North, New Zealand; e The University of Agriculture Dera Ismail Khan, Dera Ismail Khan, Pakistan

**Keywords:** Myostatin,Matrix, Metalloproteinase,preadipocyte,pig

## Abstract

Myostatin (MSTN) resulted in reduced backfat thickness in MSTN-knockout (MSTN-KO) pigs, whereas the underlying mechanism remains elusive. In this study, RNA sequencing (RNA-seq) was used to screen differentially expressed genes (DEGs) in porcine fat tissues. We identified 285 DEGs, including 4 adipocyte differentiation-related genes (ADRGs). Matrix Metalloproteinase-2/7 (MMP-2/7), fibronectin (FN), and laminin (LN) were differentially expressed in MSTN-KO pigs compared with wild-type (WT) pigs. To investigate the molecular mechanism, we treated the preadipocytes with siRNA and recombinant MSTN protein. The results indicated that MSTN increased the expression of MMP-2/7/9 and promoted the preadipocyte differentiation. To further validate the effect of MSTN on MMP-2/7/9 expression, we treated MSTN-KO PK15 cells with recombinant MSTN protein and detected the expression of MMP-2/7/9. The data showed that MSTN increases the expression of MMP-2/7/9 in PK15. This study revealed that MSTN promoted preadipocyte differentiation and provided the basis for the mechanism of fatty deposition in pigs.

## Introduction

Fat deposition is conductive to pork quality in pigs and the human health problems caused by obesity. The underlying mechanisms of fat deposition have not been fully understood. Myostatin (MSTN) is a secreted protein that acts as a negative regulator of skeletal muscle mass. MSTN is expressed almost exclusively in skeletal muscles, whereas detectable levels of myostatin RNA are also present in adipose tissues, suggesting that MSTN could play a role in regulating adipocyte differentiation [1]. In vitro, MSTN promotes adipogenesis in the multipotential C3H 10 T1/2 mesenchymal cell line [2] and inhibits adipogenesis in 3T3L1 preadipocytes [3]. In vivo, MSTN inactivation can decrease the backfat thickness in MSTN-KO pigs [4–6]. *In-vitro* and *in-vivo* studies have shown consistent results with regard to MSTN’s effect on fatty deposition. However, the effect of MSTN on adipogenesis has not been well studied.

During preadipocyte differentiation, the change in cell shape from fibroblastic morphology to rounded adipocyte is accompanied by major variations in expression and structure of several extracellular matrix (ECM) components, including collagen (CL), fibronectin (FN), and laminin (LN). The matrix metalloproteinases (MMPs) system plays an important role in these processes [7]. MMPs are involved in ECM degradation, while MMP-2/7/9 interacts with FN and LN [8–10]. FN is a key component of ECM, regulating the shape and stretch of the cells. FN degradation promotes lipid accumulation and adipogenic gene expression in porcine primary preadipocytes [11]. Furthermore, antibodies against integrin subunits markedly reduced rat preadipocyte migration on LN, another important component of ECM that plays a critical role in cell migration and other morphological aspects of preadipocyte development [12].

Previous studies have shown that MSTN, MMPs, and ECM play important roles in regulating adipocyte differentiation. We hypothesize that MSTN contributes to adipocyte differentiation through the suppression of MMPs. In this study, MSTN-KO PK15 and differentiating preadipocytes were cultured to investigate the effects of MSTN on MMPs expression. CRISPR/Cas9-mediated homologous recombination (HR) was exploited to mono-allelically or bi-allelically knock out MSTN in PK15, and then we analysed the expression of MMP-2/7/9. In preadipocytes, we used RNAi and the recombinant MSTN protein to treat the cells for loss-of-function and gain-of-function analysis. The expression of MMP-2/7/9 and preadipocyte differentiation was determined by qPCR and Western blotting. The results validated the hypothesis, laying the groundwork to reveal the mechanism of MSTN on adipogenesis and fat deposition in pigs.

## Results

### MMP-2/7 reduced in the backfat of MSTN-KO pigs

**Figure 1.**
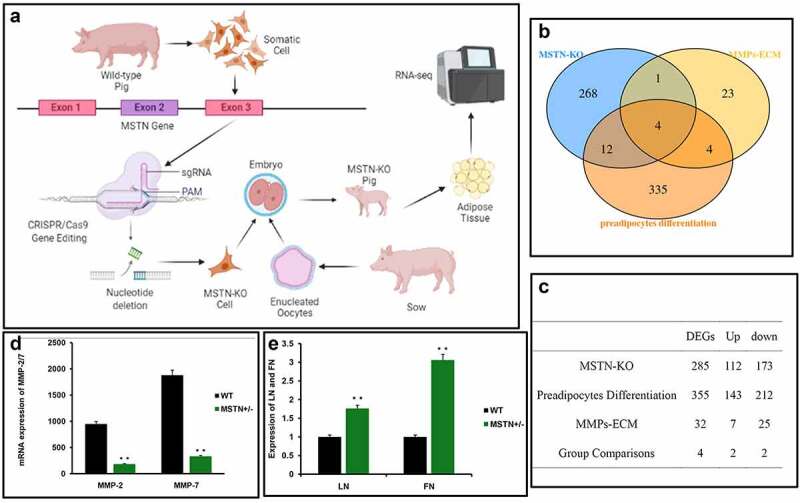
**Generation of cloned MSTN-KO pigs via DUFAS and identification of mRNA potentially involved in preadiopcytes differentiation. (a)** The schematic presentation of DUFAS-mediated HDR in pigs and RNA-seq of adipose tissue. (b) Venn diagram of DEGs in three group comparisons. (c) Summary of DEGs in MSTN-KO, MMPs-ECM, and preadipocytes differentiation comparisons. (d) Expression of MMP-2 /7 in adipose tissue of wild-type and MSTN-KO pig.

CRISPR/Cas9-mediated HR was exploited to KO allele of MSTN in pig in our previous works [4,13]. We generated cloned MSTN-KO Meishan pigs followed by RNA-sequencing of backfat tissues to profile transcriptomes of MSTN-KO and WT pigs (Figure 1a).

We found the DEGs in MSTN-KO pigs compared with the previous study of preadipocytes differentiation DEGs [13,14] (Figure 1b). The comparison between MSTN-KO and WT pigs showed 285 DEGs, of which 112 genes were upregulated and 173 genes were downregulated. Differentiated and undifferentiated preadipocytes comparison showed 355 DEGs with 143 genes were upregulated, while 212 genes were downregulated. We used a Venn diagram of three overlapping circles to represent that MSTN regulates preadipocyte differentiation through the MMPs-ECM system. Of those DEGs, only four genes were differentially expressed in MSTN-KO, preadipocytes differentiation, and MMPs-ECM comparisons (Figure 1c). Compared with the WT, MMP-2/7 were significantly reduced (Figure 1d), while FN and LN were significantly increased in the backfat of MSTN-KO pigs (Figure 1e).

### Myostatin promoted the preadipocyte differentiation by upregulating the expression of MMP-2/7/9

**Figure 2.**
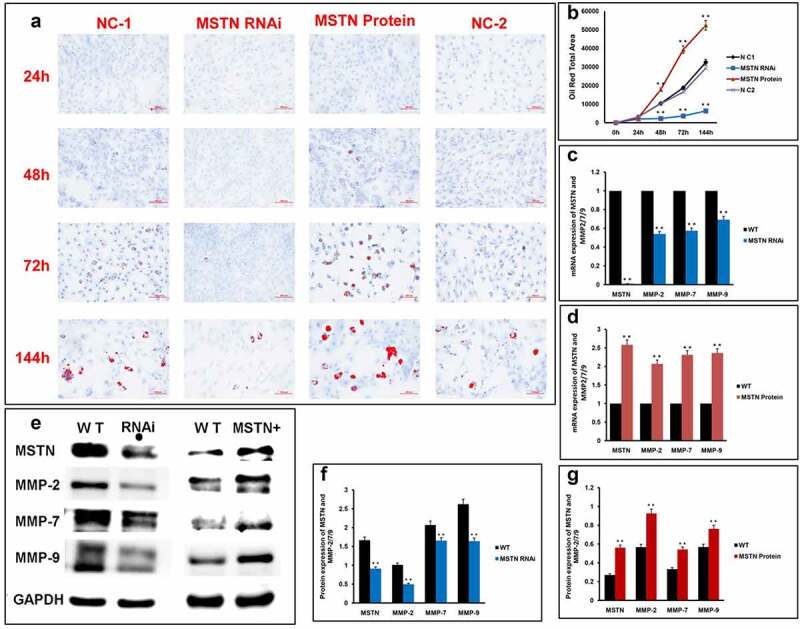
MSTN increased the expression of MMP-2/7/9 to promote the preadipocytes differentiation.

The cellular morphology change is one of the characteristics during the preadipocyte differentiation process. The recruitment of lipid droplets was another character in the process of preadipocyte differentiation. The lipid droplets were stained with Oil Red O. The intensities of the Oil Red O-staining areas of the MSTN RNAi group were significantly reduced compared with negative control from 72 to 144 h (Figure 2a, b), while that of the recombinant MSTN protein-treating group were significantly increased from 48 to 144 h (Figure 2a, b).

(a) Representative image of Oil Red O staining of preadipocytes treated with MSTN RNAi and recombinant MSTN protein from 24 to 144 h. Scale bar, 100 μm. (b) Quantification of red areas in Oil Red O staining image. (c) Transcriptions of MSTN and MMP-2/7/9 in differentiated preadipocytes treated with MSTN RNAi. *n* = 3 biologically independent samples. Differentiated preadipocyte without other treatment is the negative control. (d) Transcriptions of MSTN and MMP-2/7/9 in differentiated preadipocytes treated with recombinant MSTN protein. *n* = 3 biologically independent samples. Differentiated preadipocyte without other treatment is the negative control. (e) Expression of MSTN and MMP-2/7/9 in differentiated preadipocyte treated with MSTN RNAi and recombinant MSTN protein. *n* = 3 biologically independent samples. Differentiated preadipocyte without other treatment is the negative control. GAPDH is the internal control. (f) Quantification of expressions of MSTN and MMP-2/7/9 in differentiated preadipocyte treated with MSTN RNAi. (g) Quantification expression of MSTN and MMP-2/7/9 in differentiated preadipocyte treated with recombinant MSTN protein.

qRT-PCR and Western blotting indicated that the expression of MMP-2/7/9 genes was significantly reduced in response to MSTN RNAi (Figure 2c, e, f), while their expression in turn increased with the addition of recombinant MSTN protein in differentiated preadipocytes (Figure 2d, e g).

The data represent the mean ± SEM of three independent experiments. Compared with the control group, **p < 0.05 versus the MSTN RNAi and MSTN Protein group.

### MSTN increased the expressions of MMP-2/7/9 in MSTN-KO PK15

**Figure 3.**
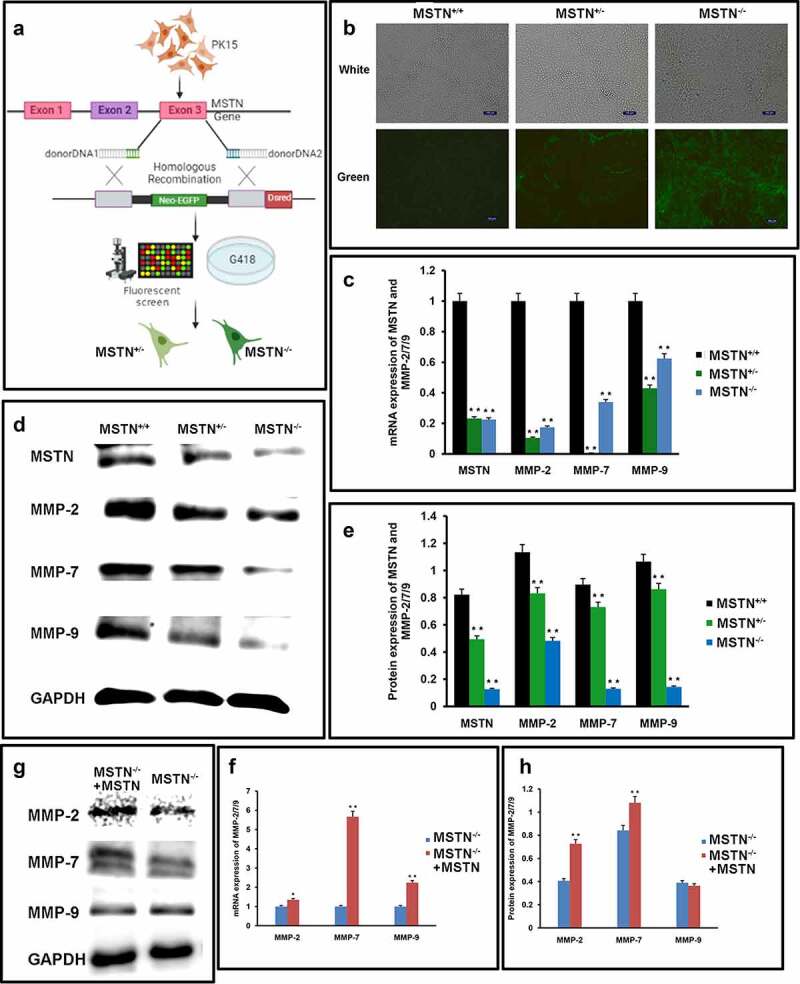
MSTN regulates MMP-2/7/9 expressions in MSTN-KO PK15.

It was refractory to perform the loss-of-function and gain-of-unction analysis in primary cells. This prompted us to apply dual fluorescence-assisted selection (DUFAS) to the targeting sites at the MSTN coding sequence in the immortal cell-line PK15 [13] (Figure 3a). The donor DNA armed with homolog immediately surrounding the 20 *nt* Cas9-editing sites were transfected with Cas9/gRNA plasmids into the PK15 cell line for MSTN targeting. CRISPR/Cas9-mediated HR was successfully achieved with KO one allele and two alleles of MSTN in PK15, named PK3108 (MSTN^±^) and L18 (MSTN^−/−^) respectively, both of which showed green fluorescence (Figure 3b). The expression of MMP-2/7/9 was significantly reduced in PK3108 and L18 cells, while expression of MSTN was significantly reduced (Figure 3c, e).

**(a)** Schematic of DUFAS-mediated HDR in PK15. (b) Representative image of a targeted cell clone by DUFAS in bright field and EGFP emission. Left, MSTN^+/+^. Middle, MSTN^±^. Right, MSTN^−/−^. Scale bar, 100 μm. (c) Transcription of MSTN and MMP-2/7/9 in PK15 (MSTN^+/+^, MSTN^±^, and MSTN^−/−^). *n* = 3 biologically independent samples. **D**. Expression of MSTN and MMP-2/7/9 in PK15 (MSTN^+/+^, MSTN^±^, and MSTN^−/−^). *n* = 3 biologically independent samples. GAPDH is the internal control. (e) Quantification of expression of MSTN and MMP-2/7/9 in PK15 (MSTN^+/+^, MSTN^±^, and MSTN^−/−^). (f) Transcript of MMP-2/7/9 in PK15 (MSTN^−/−^) treated with recombinant MSTN protein. *n* = 3 biologically independent samples. (g) Expression of MMP-2/7/9 in PK15 (MSTN^−/−^) treated with recombinant MSTN protein. *n* = 3 biologically independent samples. GAPDH is the internal control. (h) Quantification of the expression of MMP-2/7/9 in PK15 (MSTN^−/−^) treated with recombinant MSTN protein. The data represent the mean ± SEM of three independent experiments. Compared with the control group, **p < 0.05 versus the MSTN RNAi and MSTN Protein group.

To further validate that MSTN increased the expression of MMP-2/7/9, we transiently restored MSTN in the MSTN-KO PK15 and examined the expression of MMP-2/7/9. In response to the recombinant MSTN protein, the expression of MMP-2/7/9 was upregulated at both the mRNA and protein level in L18 (Figure 3f, g, and h).

## Discussion

MSTN binds to cell-surface receptors regulating fatty deposition in pigs. The MSTN protein circulates in the blood in a latent form as a full-length precursor, which is cleaved into an aminoterminal pro-peptide, and a carboxy-terminal mature region: the active form of the molecule. Active MSTN binds to its receptor activin IIB (ActRIIB) with high affinity and regulates the target genes through different signalling pathways [15,16]. MSTN leads to the phosphorylation of TGF-a specific Smads 2 and 3, which forms a complex with Smad 4 [17]. MSTN can also activate the p38 mitogen-activated protein (MAPK), Erk1/2, Wnt, and c-Jun N-terminal kinase (JNK) signalling pathways [16,18,19]. MSTN signalling can inhibit Akt phosphorylation and downregulate the IGF-1/PI3K/AKT pathway [20]. Although MSTN does not exert its action on the nuclear factor kappa B (NF-κB), NF-κB responsive elements have been found in the promoter of the FLRG, thus indirectly regulating MSTN activity [21,22]. MSTN is predominantly expressed by the muscle; our RNAseq data showed it also expressed in adipose tissue. However, the effect of MSTN on adipocyte development is still unclear.

**Figure 4.**
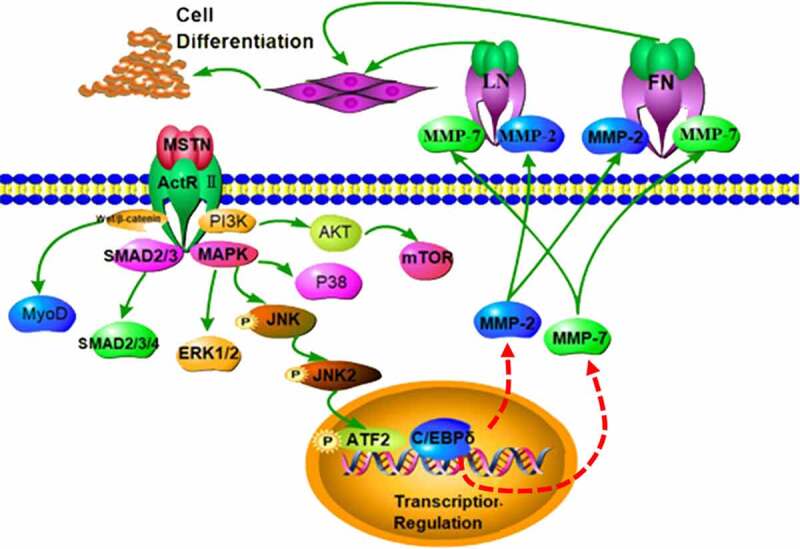
The action mode of MSTN on preadipocyte differentiation.

In the MAPK pathway, p46JNK2 has a distinct role in adipogenic differentiation presumably through the induction of ATF2-mediated CCAAT/enhancer-binding protein δ (C/EBPδ) expression in the early stage [23]. Although C/EBPs (C/EBPα and C/EBPβ) have been identified as main regulators of adipogenesis [24,25], the functional role of C/EBPδ in adipogenesis remains unclear. We hypothesize that C/EBPδ regulates the transcription of MMPs. We speculate that MSTN deficiency down-regulates the transcription of C/EBPδ, which is correlated with the MMPs and suppresses preadipocyte differentiation (Figure 4). In the MAPK pathway, phosphorylation is transferred from p46JNK2 to ATF2-mediated C/EBPδ transcription. C/EBPδ can combine with the promoter region of MMP-2/7 to enhance the transcription.

Proteinases and proteases implicated in adipogenesis include MMPs, which can degrade and break down all ECM proteins. Many MMPs are expressed by preadipocytes, especially MMP-2 and MMP-9 [26]. The MMPs inhibitors decrease adiposity in mice and consistently reduce adipogenesis in 3T3-L1 and 3T3-F442A cells [27]. The inhibitor of MMP-9 reduced adipogenesis in humans, MMP-9 might be a key factor in the regulation of human adipose tissue development [27]. Studies of transgenic mice demonstrated that adipose tissue development is affected in mice deficient in the TIMP-1 (MMP-1 inhibitor) [28]. MMPs were involved in ECM degradations, playing critical roles in morphological aspects of preadipocyte development [11,12].

MSTN regulates the ECM synthesis in cultured fibroblasts, which may have underlying functions in cell differentiation [29]. Inhibition of collagen synthesis by ethy1-3,4-dihydroxybenzoate (EDHB) decreases differentiation of bovine intramuscular preadipocyte and pig preadipocyte clonal cell lines. Deposition of collagen types IV, V, and VI was reduced by EDHB considerably more than collagen types I, II, and III in intramuscular adipocytes [30]. TGF-β1 prevents the down-regulation of FN associated with 3T3-L1 preadipocyte differentiation [31]. Some results show that FN coating not only significantly increase the yield of preadipocytes after isolation from adipose tissue but also significantly enhances the differentiation of precursor cells to mature adipocytes [32], but the major ECM component, FN, LN, and CLIV, did not affect preadipocytes differentiation when added individually to the culture medium [33].

In summary, we found backfat thickness was reduced in MSTN-KO pigs consistent with the reduction of MMP-2/7, while the FN and LN were increased. We used RNAi and recombinant MSTN protein to prove that MSTN can stimulate the expression of MMP-2/7/9 and lack of MSTN lead to the reduction of MMP-2/7/9 in differentiated preadipocytes. The mRNA and protein of MMP-2**/**7/9 were significantly reduced in PK3108 and L18 compared with PK15. It is consistent with the expression of MMP-2/7 in backfat and preadipocytes. MSTN-KO PK15 was treated with recombinant MSTN protein and showed that the expression of MMP-2**/**7/9 was significantly increased, suggesting MSTN increases the expression of MMP-2/7/9 in pigs. In terms of the tendency of MSTN to decrease MMPs expression, RNA and protein levels in the expression of MMPs in response to genetic manipulation of MSTN are not contradictory. Compared with MSTN^±^, MSTN^−/−^ reduced the MMPs protein significantly. This study showed that MSTN promoted preadipocyte differentiation, laying the groundwork to reveal the mechanism of MSTN on adipogenesis in pigs, and provided the basis for the mechanism of fatty deposition in pigs.

## Methods

### Ethics statement and animal samples

All experimental procedures involving animals were reviewed, approved, and supervised by the Animal Care Committee of the Institute of Animal Science and Veterinary Medicine, Hubei Academy of Agricultural Sciences, China. Samples of the subcutaneous fat tissues were collected, snap-frozen in liquid nitrogen, and preserved at −80°C until RNA or protein extraction.

### Plasmids, cell culture, and transfection

CRISPR/Cas9/gRNA expression plasmid px330 (Cat. No. 42,330) was obtained from Addgene. Porcine cell line PK15 was preserved by the Key Laboratory of Animal Embryo Engineering and Molecular Breeding of Hubei Province (affiliated with the Institute of Animal Science and Veterinary Medicine, Hubei Academy of Agricultural Sciences). PK15 cell line was cultured and maintained in Dulbecco’s Modified Eagle’s Medium (DMEM, Gibco) supplemented with 10% (v/v) foetal bovine serum (FBS, Hyclone) and 10 µg/ml of penicillin–streptomycin solution (Sigma-Aldrich Corp.) at 37°C in a humidified atmosphere of 5% CO_2_ and 95% air. The culture medium was refreshed every other day to ensure optimal proliferation.

Porcine preadipocytes were isolated aseptically from backfat tissue of five-day-old Meishan piglets killed via anaesthesia. Briefly, approximately 1 g of the muscle samples were collected aseptically and cut into a 1 mm^3^ cube with ophthalmic scissors in a sterile culture dish. This was followed by three rounds of washing with DPBS buffer in a 50 ml centrifuge tube and digested with 0.2 U μl-1 Type II collagenase for 2 h at 37°C in a water bath. After fully digested, the cell suspension was filtered in batches through a 74 μm cell sieve mesh and stored in new centrifuge tubes, removed tissue debris. After centrifugation at 2,000 × *g* for 5 min, the cell pellet was resuspended in DMEM/F12 medium following filtration of cell suspension through a 37 μm cell sieve mesh. After re-centrifugation, cell pellets were again resuspended with DMEM/F12 medium. Cells were then evenly inoculated in a sterilized culture flask supplied with culture medium and cultured at 37°C under 5% CO_2_ in a humidified incubator. It took approximately 2 h for subcutaneous preadipocytes to reach the adherent culture stage. The adherent cells were then washed twice with DMEM/F12 medium and then supplied with the new culture medium. The culture medium was refreshed every 3 days depending on confluency.

### RNAi and recombinant protein

Liposome transfection for siRNA was performed using Lipofectamine 3000 (Invitrogen) as described in the manufacturer’s protocol. Each group included three technical repeats. The Sequence for MSTN RNAi and negative control were designed (Table 1). We performed transfection when the cell density was 60–80%. Transfection system: 125µ L OPTI-MEM + 3.75µl Lip3000; 125l OPTI-MEM + 5 µg siRNA. The cells were collected about 48 h after transfection and determined by RT-qPCR and Western Blotting.

**Table 1.** Sequence for MSTN RNAi

siRNA Name	siRNA Sequences 5’ →3’
MSTN sense	GGU CAU GAU CUU GCU GUA ATT
MSTN antisense	UUA CAG CAA GAU CAU GAC CTT
Negative Control sense	UUC UCC GAA CGU GUC ACG UTT
Negative Control antisense	ACG UGA CAC GUU CGG AGA ATT

Recombinant MSTN protein (ab73251, Abcam) for long-term storage is to add a carrier protein (0.1% HSA or BSA), which reconstitute in sterile 20 mM HCl at 0.1 mg/ml, then further diluted to aqueous solutions according to ED_50_. MSTN recombinant protein was added to cells at concentrations of 0 ng/ml, 50 ng/ml, 100 ng/ml, and 150 ng/ml, and the concentration of 150 ng/ml was determined. Cells have collected after 0, 24, 48, 72, and 144 h, and total RNA and protein have extracted for qPCR and Western Blotting analysis.

### qPCR

cDNA synthesis was carried out using a PrimeScript RT reagent kit (Takara). qPCR was performed on a real-time thermocycler (Roche light cycle 96) with real-time PCR TB Green Premix Ex Taq II (Takara). According to the manufacturer’s instructions, the qPCR reactions were conducted in 20 μl of reaction buffer containing TB Green Premix Ex Taq polymerase II, 0.8 μl of 10 M forward and reverse primers (Table 2), 2 μl of cDNA, and 6.4 μl sterilized water. The qPCR reaction system was performed with the following steps: 2 min at 94°C followed by 45 cycles of 30 s at 94°C, 30 s at 60°C, and 30 s at 72°C, followed by a melting cycle from 55 to 95°C to assess the amplification specificity. RNA expression levels were normalized to the level of GAPDH expression. Relative expressions were calculated using the comparative 2^−∆∆CT^ method. A paired *t*-test (significant *p*-value <0.05, two-tailed) was employed to assess the significance of the difference.

**Table 2.** Primers for RT-qPCR

Genes	Primer sequences 5’ →3’	*T_m_* (°C)	Size bp	Gene Bank No.
*MMP-2*	F:TCCATGACGGAGAGGCTGACATC	60	194	NM_214192
	R:TCCATACTTCACACGCACCACTTG			
*MMP-7*	F:GCACTCACTGCCTCCATCCTTG	60	120	NM_001348795
	R:CGTTGCCTGAGGAGAGTCAAGAT			
*MMP-9*	F:TGAAGACGCAGAAGGTGGATT	60	202	NM_001038004
	R:CACTTCAGGAGGTCGAAGGTC			
*MSTN*	F:TGGCAGAGCATTGATGTGAAG	60	293	NM_214435
	R:GCAATAATCCAGTCCCATCCA			
*GAPDH*	F:GGTGAAGGTCGGAGTGAACG	60	233	NM_001206359.1
	R:CTCGCTCCTGGAAGATGGTG			

### Western blotting

Total cellular protein extracts were collected from the RIPA Lysis and Extraction Buffer (ThermoFisher). Protease inhibitor (PMSF) at a concentration of 1% was added into the RIPA buffer to avoid proteolysis. For each sample, 50 μl protein lysate collected in a 1.5 ml centrifuge tube was treated with an ice-bath for 30 min, followed by centrifugation at 10,000 × *g* at 4°C for 5 min. Protein supernatant in the tube was transferred into a new tube and stored at 4°C for further protein quantification by BCA Protein Assay Kit (P0006, Beyotime Institute of Biotechnology). 20 μg of protein extract for each sample was subjected to 12% sodium dodecyl sulphate-polyacrylamide gel electrophoresis (SDS-PAGE Gel Preparation Kit, Beyotime) and transferred to Polyvinylidene difluoride (PVDF) membrane (Bio-rad). The membranes were blocked in TBST (150 mM NaCl, 20 mM Tris-HCl at pH 8.0, 0.05% Tween 20) blocking buffer with 5% non-fat dry milk powder at room temperature for 1 h. After three washes with TBST buffer, the membranes were then incubated with primary antibodies, overnight at 4°C with shaking. After three washes in TBST buffer, blots were incubated with secondary antibodies. Protein bands were then visualized by ECL detection reagent (BeyoECL Moon, Beyotime). Band identification and quantification were conducted using a ChemiDoc™ XRS^+^ System and Image Lab Software (BioRad). The primary antibodies used for Western blotting were rabbit monoclonal antibody against the mouse MMPs (73,959, Cell Signalling Technology; dilution 1:1,000), rabbit polyclonal antibody against the mouse MSTN (TA343358, Origene; dilution 1:1,000), mouse monoclonal antibody against the human GAPDH (60,004-1-Ig, Proteintech, Wuhan, China; dilution 1:2,000), HRP-conjugated secondary antibodies were anti-mouse and anti-rabbit IgG (7076 and 7074, respectively, Cell Signalling Technology; dilution 1:2,000).

### Histology

The preadipocytes were stained with Oil Red O. Briefly, the cells were washed gently with pre-warmed DPBS twice and then fixed with 4% paraformaldehyde for 45 min. One millilitre of the ready-to-use Oil Red O solution was slowly added to cells for staining for 30 min. The colorimetric development of lipid droplets was observed under microscope and photographed. Five randomly selected fields of the Oil Red O-stained preadipocytes were quantified using the Image J software (NIH, USA).

### Statistics

Band intensities were quantified by Image J (NIH, USA). The data and results were analysed using the SPSS 15.0 software and are presented as the mean ±SEM. One-way ANOVA was used to evaluate the differences between the mean values of normally distributed data. A p value of <0.05 was considered to be statistically significant. All artworks were created using CorelDRAW Graphics (Corel Corporation, Ottawa, Canada). Phenotyping, genotyping, and RNA-seq were performed in triplicates (*n* = 3 biologically independent samples). Results are representative of multiple replicates showing similar results.

## Data Availability

The datasets presented in this study can be found in the Science Data Bank. The names is ‘Myostatin Increases the Expression of Matrix Metalloproteinase genes to Promote Preadipocytes Differentiation in Pigs’, the accession number is CSTR 31253.11.sciencedb.01347. https://www.scidb.cn/s/3i6Z3m.
